# Charlatanry—Empiricism—Quackery

**Published:** 1859-02

**Authors:** 


### *Charlatanry—Empiricism—Quackery.*

If an individual would be respected, he must respect himself; and what is true of an individual in this connection, is equally true of communities, corporations, or professions. It is a lamentable admission, that certain words which do not belong to us especially, either because of their etymology or abstract meaning, are, nevertheless, now almost exclusively appropriated to us, because in our conduct is evinced their readiest application, the whole professional life of some being a running commentary upon them. There is not any grave distinction between the graduated and ungraduated quack, and what there is, is certainly in favor of the latter. One man may use the cloak of religion in which to practice medicine; another tells you that he cures cancer and consumption. Who is the more disreputable, the liar or the hypocrite? There is less distinction still between the charlatan, whose disgusting notices, over his own signature, occupy so great a space in daily newspapers, and the physician who avails himself of the same means, but in a less manly and more insidious manner. One states that by sending a dollar to his address, you will receive an infallible cure for piles;—the other notices himself in the morning paper, in connection with an accident, which would have proved fatal, but that, connected with the unfortunate occurrence, was one so fortunate that the patient had cause to rejoice rather than mourn, for by its means he had fallen under the care of a distinguished, polished, agreeable, skillful, and eminently successful surgeon, who, by the way, had had several precisely similar cases under his care previously, all of which had been successful, not one of them had even presented an eschar which could be distinguished by the naked eye. The one states that he possesses a sure and certain remedy for stammering, bronchitis, gout and stricture;—the other is noticed by some penny-a-liner who chanced to pass his office, and who, having had his attention directed thereto, by hearing a merry laugh, was induced to inquire as to its source, and to his utter astonishment found that it proceeded from a patient upon whom an operation was being performed for a distorted condition known among medical men as Talipes Varus (vulgarly called Club-foot.) The patient was laughing because of the pleasurable sensations induced by the operation, the operator having evinced the utmost complacency throughout, in in consequennce of his familiarity with such scenes. The reporter also deems it due to suffering humanity, and to persons with similar deformities in particular, to state that in all similar cases of this gentleman upon which he had operated, no rigidity whatever remained, but the feet operated upon, were immediately and ever afterward, as limber as lambs' tails.

We do not distinguish between these modes of advertising, except to admire the bold, unblushing effrontery of the one, who, over his signature, promises to cure, when compared with the duplicity of the other.

We know that the parties named are not always responsible for the comments made, but some of their professional friends frequently are; and it is to denounce such acts of mistaken favor on their part, that we write: for while lowering the subject of their eulogy in the estimation of his peers, they also debase the profession before the community, and reduce it to a level with the most arrant quackery.—*Louisville, Medical News.*

